# Predictors for Using Electricity During Hysteroscopic Removal of Retained Products of Conception

**DOI:** 10.3390/jcm14217587

**Published:** 2025-10-26

**Authors:** Liat Mor, Tzvi Leibowitz, Emilie Ben-Ezry, Ram Kerner, Ran Keidar, Eran Weiner, Ron Sagiv, Ohad Gluck

**Affiliations:** 1 Department of Obstetrics & Gynecology, The Edith Wolfson Medical Center, Holon 58100, Israel; emilie.ben.ezry@gmail.com (E.B.-E.); ram_kerner@yahoo.com (R.K.); keidaron144@gmail.com (R.K.); eranw@wmc.gov.il (E.W.); sagivr@wmc.gov.il (R.S.); ohadg@wmc.gov.il (O.G.); 2 Faculty of Medicine, Tel Aviv University, Tel Aviv 69978, Israel; tzvi.leibo@gmail.com

**Keywords:** retained products of conception, hysteroscopy, electrosurgery, operative hysteroscopy, Doppler ultrasound

## Abstract

**Background:** Retained products of conception (RPOC) can be managed via hysteroscopic removal using mechanical or electrosurgical techniques. Electrosurgery introduces greater technical complexity and may reflect more adherent or vascular tissue, yet preoperative predictors for its necessity remain poorly defined. **Objective:** The objective of this study was to evaluate clinical outcomes and identify preoperative predictors associated with the use of electrosurgery during hysteroscopic removal of RPOC. **Methods:** In this retrospective cohort study conducted at a single tertiary center, we reviewed 551 cases of hysteroscopic RPOC removal performed between January 2008 and December 2022. Patients were categorized based on intraoperative use of electrosurgical instruments. Clinical, sonographic, and operative data were compared between groups. Multivariate logistic regression was used to identify independent predictors of electrosurgical use. **Results:** Electrosurgical intervention was required in 84 patients (15.2%). Compared with those treated without electricity, these patients were older (33.2 ± 6.4 vs. 31.2 ± 5.8 years, *p* = 0.004), more likely to be smokers (15.4% vs. 8.1%, *p* = 0.033), and had higher rates of prior hysteroscopy (5.9% vs. 1.0%, *p* = 0.002). Electrosurgical use was more common following vaginal delivery than abortion (57.1% vs. 24.8%, *p* < 0.001), particularly when manual placental removal was performed (23.8% vs. 5.7%, *p* < 0.001). Larger RPOC size and positive Doppler flow were also associated with the use of electrosurgery. On multivariate analysis, maternal age, postpartum RPOC, manual placental removal, and Doppler vascularity remained independent predictors. No significant differences were observed in short-term postoperative complications. **Conclusions:** Older age, postpartum RPOC, manualysis, and vascularity on ultrasound are preoperative predictors for the need of electrosurgical intervention during hysteroscopic removal of RPOC. Identifying these factors may improve surgical planning and patient counseling. Future prospective studies incorporating advanced hysteroscopic technologies are warranted.

## 1. Introduction

Retained products of conception (RPOC) refer to intrauterine remnants of trophoblastic or fetal tissue that persist following delivery or abortion. The prevalence of RPOC varies depending on gestational age and mode of pregnancy termination, ranging from 0.5% following term delivery to as high as 36% following first-trimester pregnancy loss [1,2]. Risk factors include preterm birth, advanced maternal age, previous cesarean delivery, prior uterine surgery, and complicated obstetric history [1,3]. RPOC may result from incomplete placental detachment, abnormal placental implantation, or impaired uterine involution, often leading to persistent vascularized tissue adherent to the endometrium or myometrium [4].

RPOC might be associated with severe short- and long-term complications including excessive bleeding, endometritis, infertility and subsequent abnormal placentation [5,6,7]. Although conservative or medical approaches may be appropriate in selected cases, these strategies have been linked to higher rates of incomplete evacuation and often result in delayed surgical intervention [8,9]. Traditionally, dilation and curettage (D&C) was the standard surgical method for RPOC management. However, operative hysteroscopy is now preferred due to its higher success rates and lower incidence of intrauterine adhesions and reproductive sequelae [5,10,11].

Hysteroscopic removal is typically performed using mechanical techniques, such as “cold-loop” excision. However, in cases of firm tissue adherence or increased vascularity, electrosurgery may be required. This introduces technical complexity and has been associated with higher rates of complications, including bleeding, uterine perforation, and intrauterine adhesions [12,13]. While it is unclear whether electrosurgery itself increases complication risk or whether it reflects more severe underlying pathology, the decision to use electricity affects operative planning. Additionally, procedures requiring electrosurgery often demand general anesthesia, greater surgical expertise, and equipment not readily available in office-based settings. Despite these clinical implications, there is a paucity of data identifying preoperative factors that predict the need for electrosurgical intervention.

Therefore, the objective of this study was to compare the outcomes of hysteroscopic RPOC removal performed with versus without electrosurgery and to identify clinical and sonographic predictors associated with the need for energy use. Anticipating this requirement preoperatively may support tailored procedural planning and optimize patient care.

## 2. Methods

This was a retrospective cohort study conducted at a single tertiary academic medical center. We reviewed the records of all patients who underwent operative hysteroscopy for the removal of RPOC between January 2008 and December 2022. This study was approved by the institutional ethics committee (Approval No. 0018-23-WOMC). During the preparation of this manuscript, the authors used Grammarly v1.2.207.1776 to improve language and readability. After using this tool, the authors reviewed and edited the content as needed and take full responsibility for the final version.

### 2.1. Study Population

Eligible participants included women who underwent hysteroscopic removal of RPOC following spontaneous pregnancy loss before 24 weeks’ gestation (medically or surgically managed), induced abortions of any type, or delivery (vaginal or cesarean) after 24 weeks’ gestation. Patients were referred to hysteroscopy following suggestive imaging with or without associated symptoms of irregular bleeding. Patients underwent transvaginal ultrasound with color Doppler imaging prior to the hysteroscopic procedure, in accordance with departmental protocol to confirm the presence, size, and vascularity of RPOC. Sono-hysterography was not routinely performed. Patients were excluded if they underwent D&C rather than hysteroscopy or if relevant data were missing. Patients who presented during the first postpartum month with excessive vaginal bleeding and RPOC was suspected were not candidates for hysteroscopy due to impaired visualization and were therefore treated with D&C instead. Detailed data on the total number of patients treated with D&C during the study period were not consistently available; therefore, these cases were excluded from this analysis. All patients underwent hysteroscopy 2–4 weeks after abortion or 4–6 weeks postpartum, based on departmental timing protocols.

### 2.2. Surgical Technique

All procedures were performed under general anesthesia according to departmental policy to ensure complete uterine distension, optimal visualization, and patient immobility. A consistent team of five senior gynecologic surgeons performed all procedures in this cohort, each with a minimum of five years’ experience in operative hysteroscopy. This ensured procedural consistency and minimized variability in technique and judgment regarding electrosurgical use. After cervical dilation, a 10 mm bipolar resectoscope (VersaPoint, Ethicon Inc., Raritan, NJ, USA) was used. Normal saline served as the distension medium, maintained at a pressure of 90–110 mmHg. The initial approach aimed to remove RPOC using a “cold-loop” technique: gentle, mechanical scraping of the uterine wall to detach the tissue. Electrosurgery (bipolar loop, coagulation mode, with a default power setting of 100 W) was employed when mechanical resection was ineffective, typically due to tissue adherence, vascularity, or unclear tissue planes. While department-wide guidance encouraged cold-loop excision as first-line, the final decision to employ electrosurgery was made by the attending surgeon based on intraoperative judgment. Cases in which electricity was used solely for hemostatic purposes were excluded. No hysteroscopic tissue morcellators were used during the study period. All excised specimens were submitted for histopathological confirmation.

### 2.3. Data Collection

Clinical and demographic data were extracted from electronic records, including age, gravidity, parity, smoking status, history of uterine surgeries (hysteroscopy, cesarean, D&C), and indication for hysteroscopy (abortion or postpartum). Sonographic variables included RPOC diameter, location, and vascularity (as assessed by color Doppler imaging). Procedural variables included need for electrosurgery, estimated blood loss (EBL), intraoperative complications, and RPOC location.

### 2.4. Statistical Analysis

Comparisons between patients treated with versus without electrosurgery were conducted using the student’s *t*-test for normally distributed continuous variables, the Mann–Whitney U test for non-normally distributed variables, and the chi-square or Fisher’s exact test for categorical data. Multivariate logistic regression was performed to identify independent predictors of electrosurgical use, incorporating variables found to be significant on univariate analysis (*p* < 0.05). All analyses were conducted using SPSS version 29 (IBM Corp., Armonk, NY, USA), and statistical significance was set at *p* < 0.05.

## 3. Results

During the time of study, a total of 551 patients underwent hysteroscopic removal of RPOC, of whom 84 (15.2%) required the use of electrosurgical instruments and were included in the study group which necessitated electrical removal, and the remaining 467 (84.8%) patients served as the control group with no electrical removal.

Table 1 presents the demographic characteristics of the groups. Patients in the electric removal group were significantly older (33.2 ± 6.4 vs. 31.2 ± 5.8 years, *p* = 0.004) and had higher rates of smoking and prior hysteroscopies (15.4% vs. 8.1%, *p* = 0.033 and 5.9% vs. 1.0% *p* = 0.002, respectively). However, rates of past abdominal surgeries, past D&Cs, gravidity, and parity did not differ between the groups.

Table 2 demonstrates the pre-intervention symptoms and RPOC characteristics of the study groups. Electrical use was significantly more common in patients with RPOC following NVD, compared to abortion (57.1% vs. 24.8% *p* < 0.001). Of the patients who had postpartum RPOC, those who underwent manualysis (manual removal of the placenta) had a significantly higher rate of electrical use during hysteroscopy (23.8% vs. 5.7%, *p* < 0.001). Furthermore, ultrasound findings in patients requiring electricity revealed larger RPOC diameters (19.8 ± 10.9 vs. 16.8 ± 7.3 mm, *p* = 0.003) and higher rates of positive Doppler flow (47.6% vs. 25.9%, *p* = 0.037) compared to controls.

Intraoperatively, electric removal was more commonly used in RPOCs located on the anterior uterine wall (46.4% vs. 15.6%, *p* = 0.017). In addition, estimated blood loss was higher (28.4 ± 39.9 vs. 12.3 ± 18.7 mL, *p* < 0.001). (Table 3).

Table 4 demonstrates the results of a multivariate regression analysis exploring independent risk factors associated with the use of electricity during hysteroscopy for RPOC removal. Increasing patient age (OR 1.06, 95% CI 1.01–1.11, *p* = 0.007), RPOC following NVD (OR 2.22, 95% CI 1.13–4.35, *p* = 0.020), manualysis (OR 2.20, 95% CI 1.00–4.82, *p* = 0.048), and positive Doppler flow (OR 1.73, 95% CI 1.02–3.00, *p* = 0.049) were all found to be significantly and independently associated with the need for electrocoagulation.

## 4. Discussion

This study evaluated the background, clinical, and sonographic characteristics of patients undergoing hysteroscopic removal of RPOC following abortion or delivery, with a focus on identifying factors associated with the use of electricity during RPOC removal. We found that older patient age, patients who had RPOC following delivery (compared to abortion), especially those who underwent manualysis, and the presence of vascularization (according to Doppler), are independent risk factors for the need of electricity use during operative hysteroscopy. Interestingly, the size of the RPOC on preprocedural US was not independently associated with the use of electricity.

While previous studies have compared complications and success rates of hysteroscopic versus curettage techniques for RPOC management [5,6,14], few have specifically examined which preoperative clinical or sonographic factors predict the need for electrosurgical intervention. Identifying such predictors is clinically relevant because the use of electricity implies a more complex, adherent, or vascular pathology requiring general anesthesia and advanced equipment. Anticipating these cases preoperatively may improve patient counseling, surgical planning, and referral decisions, particularly in centers where office hysteroscopy is the first line approach.

Our findings align with previous research, which identifies more vascular RPOCs as more technically challenging and potentially requiring more advanced surgical tools. Rein et al. have shown that RPOC following term deliveries, particularly NVD, tend to be more vascular and deeply implanted, increasing the risk of intraoperative bleeding and requiring electrosurgical dissection [15]. The increased prevalence of positive Doppler flow among patients requiring surgery in our study supports this, suggesting that vascularity may be an important preoperative predictor of surgical complexity. Similarly, Hooker et al. observed that vascularized RPOC, as identified by color Doppler ultrasound, were more likely to require active surgical management and had higher complication rates, underscoring the importance of sonographic evaluation in surgical planning [5].

The significant association between manualysis and RPOC is largely evident [16,17]. Logically, a defective third stage requiring manual removal of the placenta suggests a pathological and adherent placental invasion, possibly explaining the necessity of electrosurgical tools. The association between postpartum RPOC and the need for electricity during hysteroscopy is also intuitive. A meta-analysis by Vitale et al. previously showed higher rates of incomplete hysteroscopic resection of RPOC following delivery compared to post-abortion evacuation [14], in line with our findings of increased electrical use in post-NVD cases compared to post-abortion hysteroscopies.

Surprisingly, another predictor of electrocoagulation use during hysteroscopy, as highlighted in our results, is advanced patient age. However, this observation is not supported by previous studies and contributes slightly to the overall risk. Several mechanisms may underline this relationship. Older patients are more likely to have a history of uterine procedures, subclinical fibrosis, or reduced endometrial compliance, which can make mechanical removal of RPOC more technically challenging. Furthermore, it should be noted that patient age may not merely be a demographic variable, but also a reflection of increased accumulated risk for other confounders, which may influence surgical difficulty and the need for energy-based interventions, such as prior abdominal surgeries or uterine procedures. Although these factors did not differ between patients with vs. without electrocoagulation use in a univariate analysis, the synergistic effect of these factors may be reflected in advanced age, which may contribute to this indirect effect.

Finally, although RPOC size was significantly greater in patients requiring electrosurgery in univariate analysis, it did not remain an independent predictor in the multivariate model. Contrary to our findings, several previous studies have highlighted the association between sonographic measurements of RPOC and success rate of RPOC removal [18,19,20]. However, these studies focused on the feasibility of office hysteroscopy without anesthesia. Since the size of the RPOC may directly affect the length of the procedure and therefore implications on patient satisfaction and pain, the size may be an indirect confounder in these studies. Our results suggest that the effect of size may be attenuated by other factors, such as vascularity, postpartum status, or prior manual removal of the placenta, which more directly reflect tissue adherence and surgical complexity. In other words, larger RPOC are often more vascular or associated with postpartum cases, and it may be these characteristics, rather than size per se, that drive the need for electrosurgical intervention.

This study has several strengths. It represents a large, single-center cohort with detailed clinical, imaging, and surgical data. The standardized surgical technique and consistent documentation allowed for meaningful comparisons. Finally, this study is the first to identify the independent risk factors associated with the use of electricity during hysteroscopy. As this intervention lengthens the procedure, and requires special equipment and anesthesia as well as a skilled surgeon, it is crucial to detect these patients in advance in order to spare them inappropriate and repeated interventions and to refer them to the necessary procedure accordingly.

However, this study is not without limitations. First, its retrospective design might contribute to selection and documentation biases. Second, this is a mixed cohort including cases of RPOC following abortions and deliveries, which may obscure the results. However, appropriate univariate and multivariate analyses were performed to assess the independent association of each RPOC subgroup with the need for electrosurgical intervention. Additionally, though the use of a hysteroscopic tissue retrieval device is gaining popularity and may be appropriate for RPOC evacuation in a “see and treat” setting without electrosurgery, all procedures in this cohort were performed using a resectoscope. However, since traditional resectoscopic hysteroscopy remains the standard approach in many tertiary centers, particularly for large or vascular RPOC, our findings remain clinically applicable to settings where energy-based resection is still routinely performed. Moreover, the surgeon’s subjective judgment may influence the decision to use electrosurgery, and Doppler flow assessment was not performed in a standardized protocol, which may have affected its predictive accuracy. Finally, long-term reproductive outcomes were not assessed, which limits conclusions regarding fertility implications. Previous studies have reported favorable fertility and pregnancy results following hysteroscopic management of RPOC compared to blind curettage with lower rates of intrauterine adhesions and high subsequent conception rates following hysteroscopic treatment [5,14]. Future prospective research should evaluate whether the use of electrosurgery specifically influences reproductive outcomes or placentation abnormalities.

Despite these limitations, our findings highlight the potential value of preoperative assessment in tailoring surgical strategy. Identifying patients at higher risk of requiring electrosurgery may prompt appropriate resource allocation, anesthesia planning, and referral to operative settings equipped for more complex hysteroscopic interventions. This is particularly relevant in ambulatory or office-based settings where cold-loop excision is preferred, and energy use is often impractical.

Future research should aim to prospectively validate these predictors and to evaluate the impact of alternative hysteroscopic modalities, such as mechanical morcellators, which may facilitate tissue removal in highly vascular or adherent RPOC without requiring electrosurgery. Comparative studies of surgical techniques, as well as evaluation of long-term reproductive outcomes, are also warranted.

## Figures and Tables

**Table 1:** Baseline characteristics of the study groups.

	Electric Removal (n = 84)	No Electric Removal (n = 467)	*p*-Value
Patients’ age (years)	33.2 ± 6.4	31.2 ± 5.8	**0.004**
Past abdominal surgeries	21 (25.0)	79 (16.9)	0.078
Smoking	13 (15.4)	38 (8.1)	**0.033**
Gravidity	2 [1–20]	2 [1–12]	0.904
Parity	2 [0–5]	1 [0–7]	0.574
Nulliparity	15 (17.8)	82 (17.5)	1.0
Prior hysteroscopy	5 (5.9)	5 (1.0)	**0.002**
Prior D&C	21 (25.0)	70 (14.9)	0.407

Continuous variables are presented as mean ± SD and categorical variables as n (%). Values in bold are statistically significant.

**Table 2:** Pre-intervention symptoms and RPOC characteristics of the study groups.

	Electric Removal (n = 84)	No Electric Removal (n = 467)	*p*-Value
RPOC following NVD	48 (57.1)	116 (24.8)	**<0.001**
RPOC following CS	5 (5.9)	19 (4.0)	0.439
RPOC following medical treatment for MA	10 (11.9)	88 (18.8)	0.124
RPOC following D&C for MA	8 (9.5)	60 (12.8)	0.390
RPOC following medical treatment for AA	4 (4.7)	24 (5.1)	0.882
RPOC following D&C for AA	9 (10.7)	28 (5.9)	0.113
Manual removal of the placenta	27 (32.1)	44 (9.4)	**<0.001**
Symptoms and preoperative assessment			
Irregular bleeding	41 (48.8)	187 (40.0)	0.260
Abdominal pain	4 (4.7)	9 (1.9)	0.116
Infection	4 (4.7)	4 (0.8)	0.466
Baseline hemoglobin	12.2 ± 1.3	12.3 ± 1.2	0.655
Diagnosed postpartum hemorrhage	5 (5.9)	19 (4.0)	0.439
Largest RPOC diameter on ultrasound (mm)	19.8 ± 10.9	16.8 ± 7.3	**0.003**
Presence of blood flow by US-doppler	40 (47.6)	121 (25.9)	**0.037**

Continuous variables are presented as mean ± SD and categorical variables as n (%). Values in bold are statistically significant.

**Table 3:** Surgical and post-surgical outcomes.

	Electric Removal (n = 84)	No Electric Removal (n = 467)	*p*-Value
RPOC location–anterior wall	39 (46.4)	73 (15.6)	**0.017**
RPOC location–posterior wall	18 (21.4)	101 (21.6)	0.065
RPOC location–uterine fundus	15 (17.8)	73 (15.6)	0.315
RPOC location–uterine horn	6 (7.1)	37 (7.9)	0.236
Duration of hysteroscopy (minutes)	19.5 ± 11.0	12.6 ± 5.3	0.192
Estimated blood loss	28.4 ± 39.9	12.3 ± 18.7	**<0.001**
Intraoperative complications	2 (2.3)	2 (0.4)	0.053

Continuous variables are presented as mean ± SD and categorical variables as n (%). Values in bold are statistically significant.

**Table 4:** Multivariate linear regression analyses evaluating the risk factors to use of electricity during hysteroscopy for RPOC removal.

	OR	95% Confidence Interval	*p*-Value
Age	**1.06**	**1.01–1.11**	**0.007**
Smoking	1.29	0.58–2.87	0.531
Nulliparity	1.59	0.75–3.37	0.223
Prior hysteroscopy	2.65	0.64–10.97	0.177
RPOC following delivery (compared to abortion)	**2.22**	**1.13–4.35**	**0.020**
Manualysis	**2.20**	**1.00–4.82**	**0.048**
Largest RPOC diameter on ultrasound (mm)	1.02	0.99–1.06	0.144
Presence of blood flow by US-Doppler	**1.73**	**1.02–3.00**	**0.049**

Results in bold are statistically significant.

## Data Availability

The datasets used and analyzed during the current study are available from the corresponding author upon a reasonable request. Please contact the corresponding author at liatmo@wmc.gov.il for further information.
